# Supplementary material for: Mutations in SMARCB1 and in other Coffin–Siris syndrome genes lead to various brain midline defects
Source: Nat Commun. 2019 Jul 4;10:2966. doi: 10.1038/s41467-019-10849-y (PMC6609698; doi:10.1038/s41467-019-10849-y)
Supplement: Supplementary file 1 — Supplementary Information [file 41467_2019_10849_MOESM1_ESM.pdf]

Supplementary Information

Mutations in *SMARCB1* and in other Coffin-Siris syndrome genes lead to various brain midline defects

Filatova et al., Nature Communications 2019

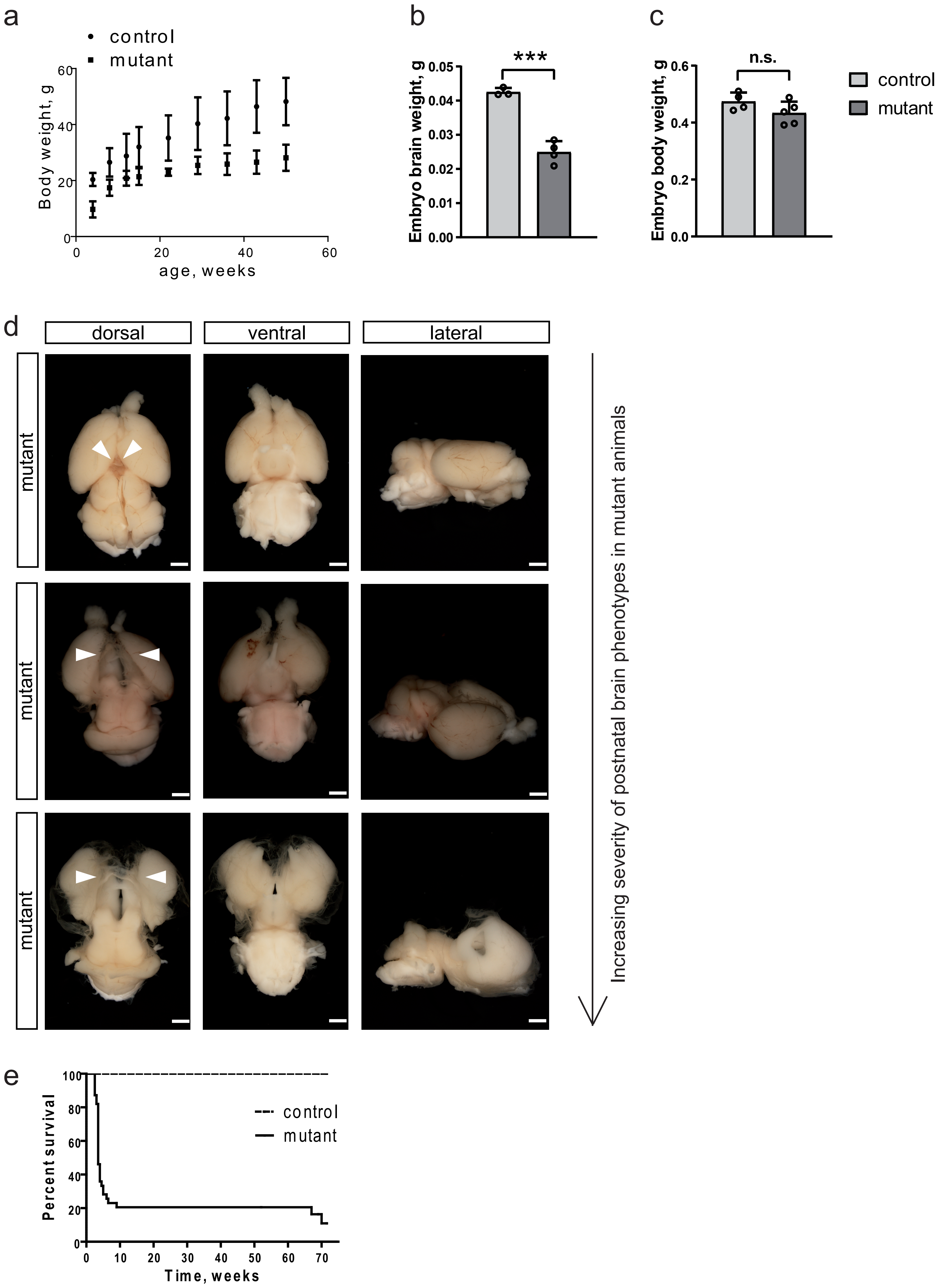
a
control
mutant
Body weight, g
age, weeks
b
Embryo brain weight, g
***
c
Embryo body weight, g
n.s.
control
mutant
d
dorsal
ventral
lateral
mutant
mutant
mutant
Increasing severity of postnatal brain phenotypes in mutant animals
e
Percent survival
Time, weeks
control
mutant

Supplementary Fig. 1

Brain, body weight and life span of animals, and spectrum of brain phenotypes in mutant mice. (**a**) Postnatal mutant mice (seven animals) have a lower body weight than control mice (ten animals). The mean plus standard deviation is displayed. (**b**) The brain weight of E14.5 mutant embryos (four animals) is significantly reduced compared to control embryos (three animals). (**c**) Control (four animals) and mutant (five animals) E14.5 embryos do not show any significant difference in body weight. All bars display the mean with standard deviations. p-values (unpaired two-tailed Student's t-tests): n.s. not significant, *** $p \leq 0.001$.
(**d**) Photographs of brains from mutant mice with increasing phenotypic severity. Arrowheads in the left panel point to regions where a midline fusion of the cerebral hemispheres is missing. The age of the mutant animals is P44 (upper panel), P23 (middle panel), and P19 (lower panel). Scale bars: 1 mm. (**e**) About 80% of mutant mice die before week 9. 7 control and 39 mutant animals were analyzed. Source data are provided as a Source Data file.

a

No.	Parents	Offspring					
		Smarcb1 $^{inv/inv}$ *NesCre* $^{+/-}$	*Smarcb1* $^{+/inv}$ *NesCre* $^{+/-}$	*Smarcb1* $^{inv/inv}$ *NesCre* $^{-/-}$	*Smarcb1* $^{+/inv}$ *NesCre* $^{-/-}$	*Smarcb1* $^{+/+}$ *NesCre* $^{+/-}$	*Smarcb1* $^{+/+}$ *NesCre* $^{-/-}$
1	♂ *Smarcb1* $^{+/inv}$ *NesCre* $^{-/-}$<br>♀ *Smarcb1* $^{+/inv}$ *NesCre* $^{+/-}$	0/7	1/7	3/7	0/7	2/7	1/7
2	♂ *Smarcb1* $^{+/inv}$ *NesCre* $^{+/-}$<br>♀ *Smarcb1* $^{+/inv}$ *NesCre* $^{-/-}$	2/10	2/10	3/10	1/10	1/10	1/10
3	♂ *Smarcb1* $^{+/inv}$ *NesCre* $^{+/-}$<br>♀ *Smarcb1* $^{inv/inv}$ *NesCre* $^{-/-}$	1/6	2/6	2/6	1/6		
4	♂ *Smarcb1* $^{+/inv}$ *NesCre* $^{+/-}$<br>♀ *Smarcb1* $^{inv/inv}$ *NesCre* $^{-/-}$	0/7	2/7	1/7	4/7		

b

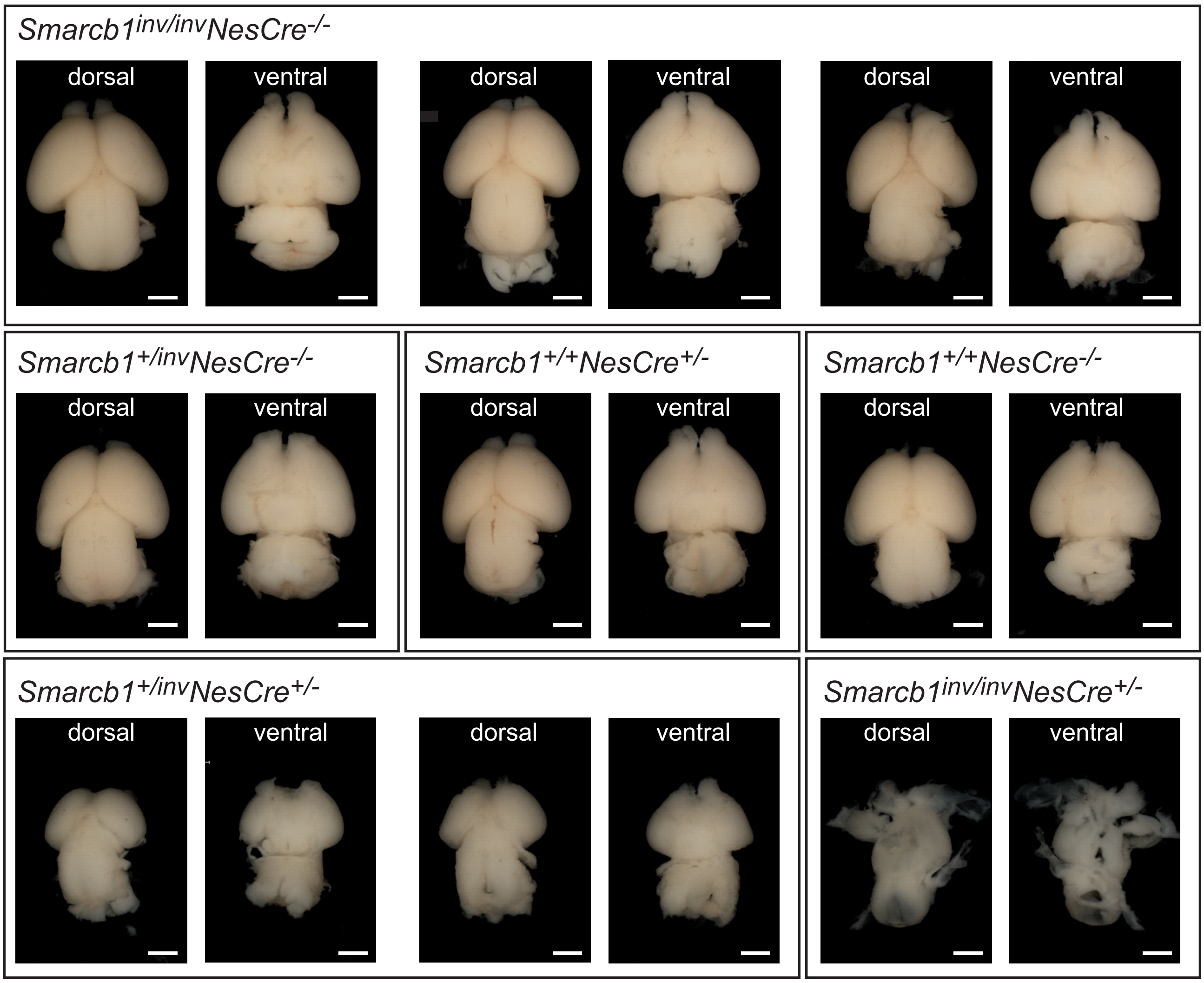

Supplementary Fig. 2

Breeding outcomes with parental mice of different genotypes. (**a**) Genotypes of mice without any obvious brain abnormalities are highlighted in grey. The age of the analyzed offspring was P24 (breeding 1), E18 (breeding 2), E18.5 (breeding 3), and E15 (breeding 4). (**b**) Photographs of E18 brains isolated from mice generated by crossing a *Smarcb1* $^{+/inv}$ *NesCre* $^{+/-}$ male with a *Smarcb1* $^{+/inv}$ *NesCre* $^{-/-}$ female (breeding 2). Dorsal (left hand side) and ventral (right hand side) views are shown. Scale bars: 1 mm.

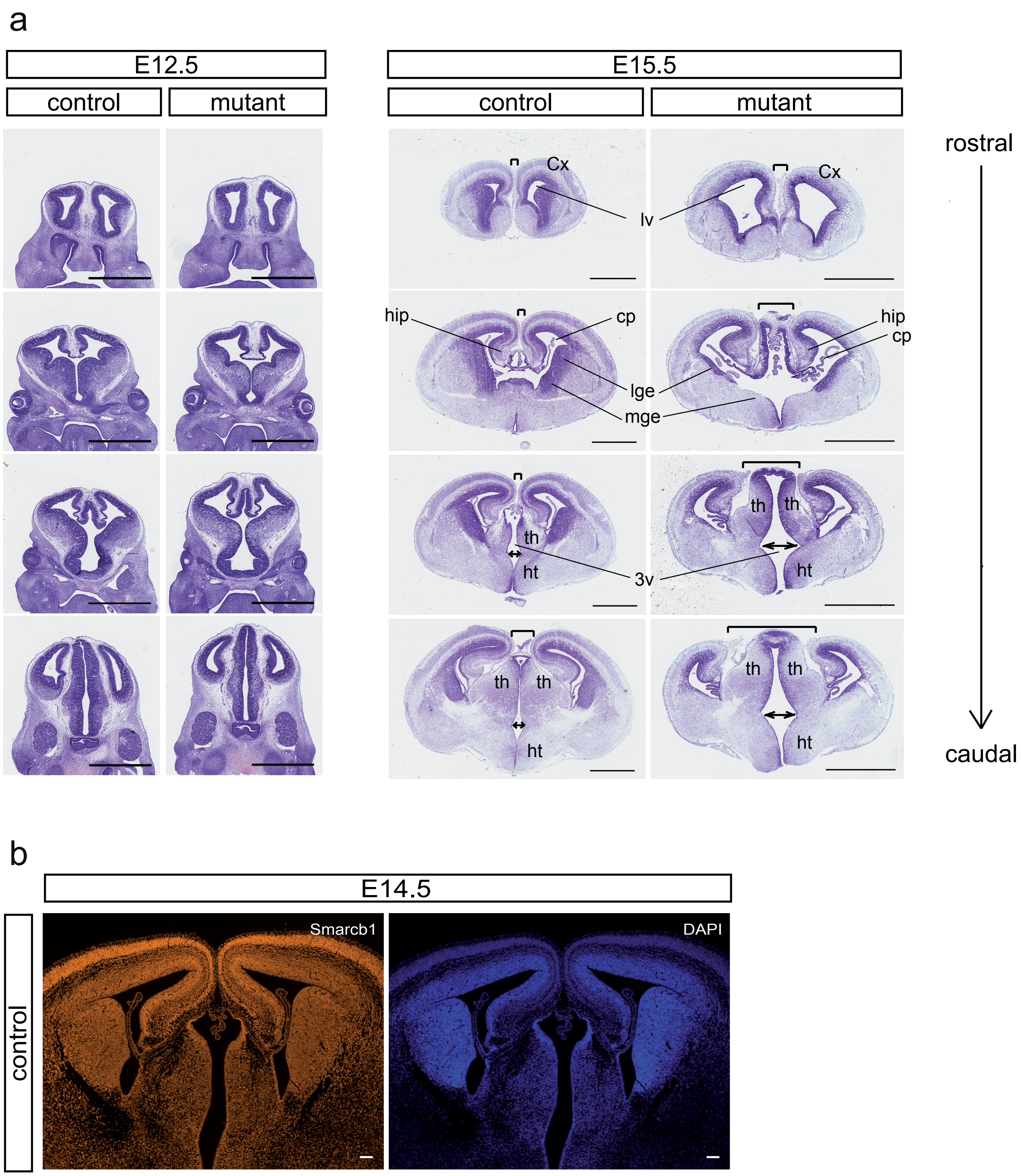

Supplementary Fig. 3

Nissl-stained and immunostained embryonic brain sections. (**a**) No obvious morphological defects are seen on Nissl-stained coronal brain sections from E12.5 mutant embryos, while an increased interhemispheric distance (brackets) and increased distance between the diencephalic halves (double arrows) is present in E15.5 mutant animals. Scale bars: 1 mm. Cx: cortex, lv: lateral ventricle, hip: hippocampus, cp: choroid plexus, lge: lateral ganglionic eminence, mge: medial ganglionic eminence, th: thalamus, ht: hypothalamus. (**b**) Smarcb1 immunostaining of a coronal forebrain section from a E14.5 control embryo. Scale bars: 100 µm.

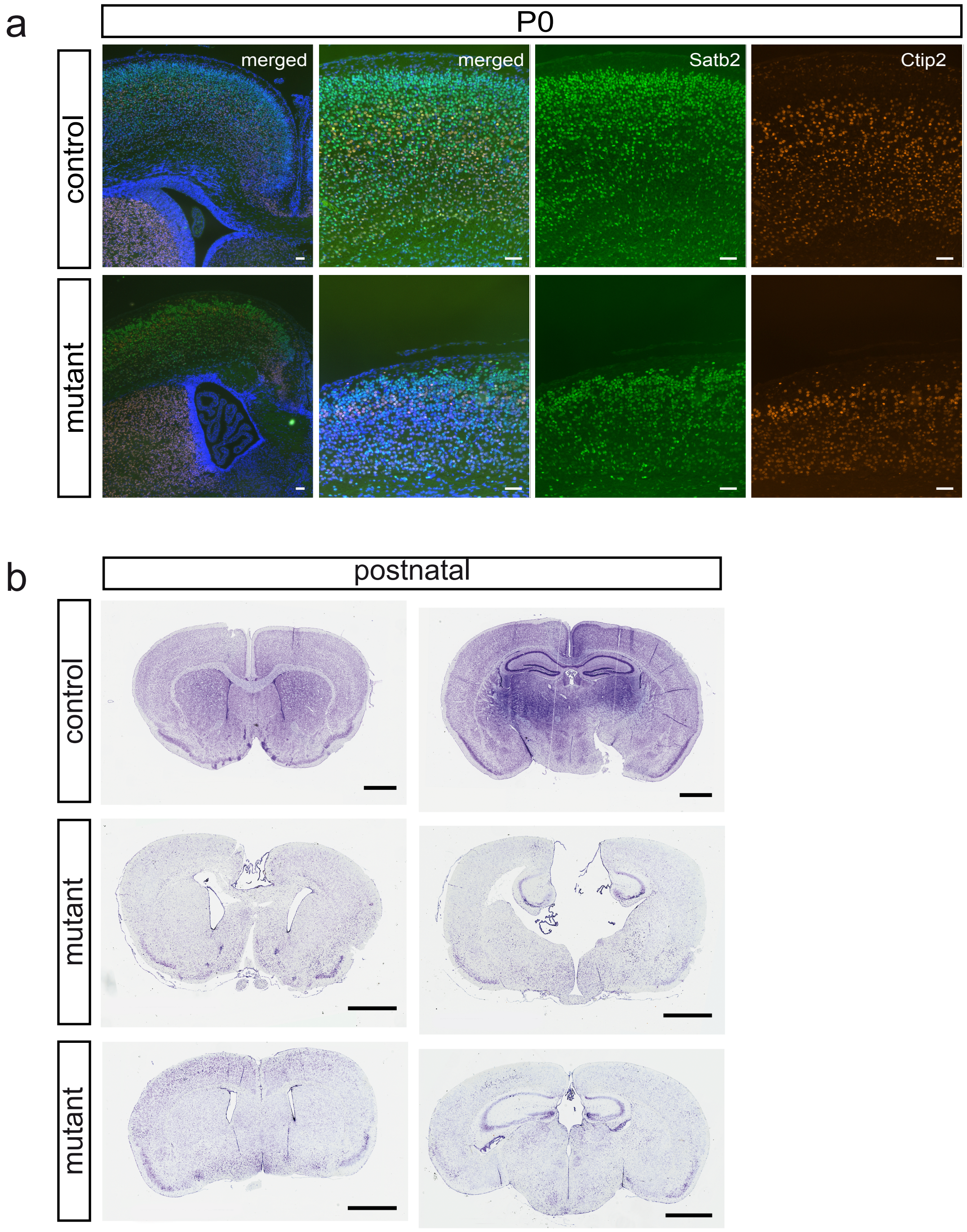

Supplementary Fig. 4

Fewer neurons in mutant mice, but Satb2-positive commissural neurons are present. (**a**) Immunostainings of P0 coronal brain sections show that Satb2-positive neurons, which give rise to the majority of callosal axons, are present in the upper cortical layers of mutant mice. Ctip2- (red) and Satb2- (green) positive neurons of lower and upper cortical layers are shown, respectively. DAPI-positive nuclei are stained in blue. The two middle and the right panels show higher magnifications of the region depicted in the left panel. Scale bars: 100 µm. (**b**) Nissl staining of coronal sections from 3-4-week-old animals shows a reduced number of neurons in mutant mice. One control and two mutant mice are presented. Scale bars: 1 mm.

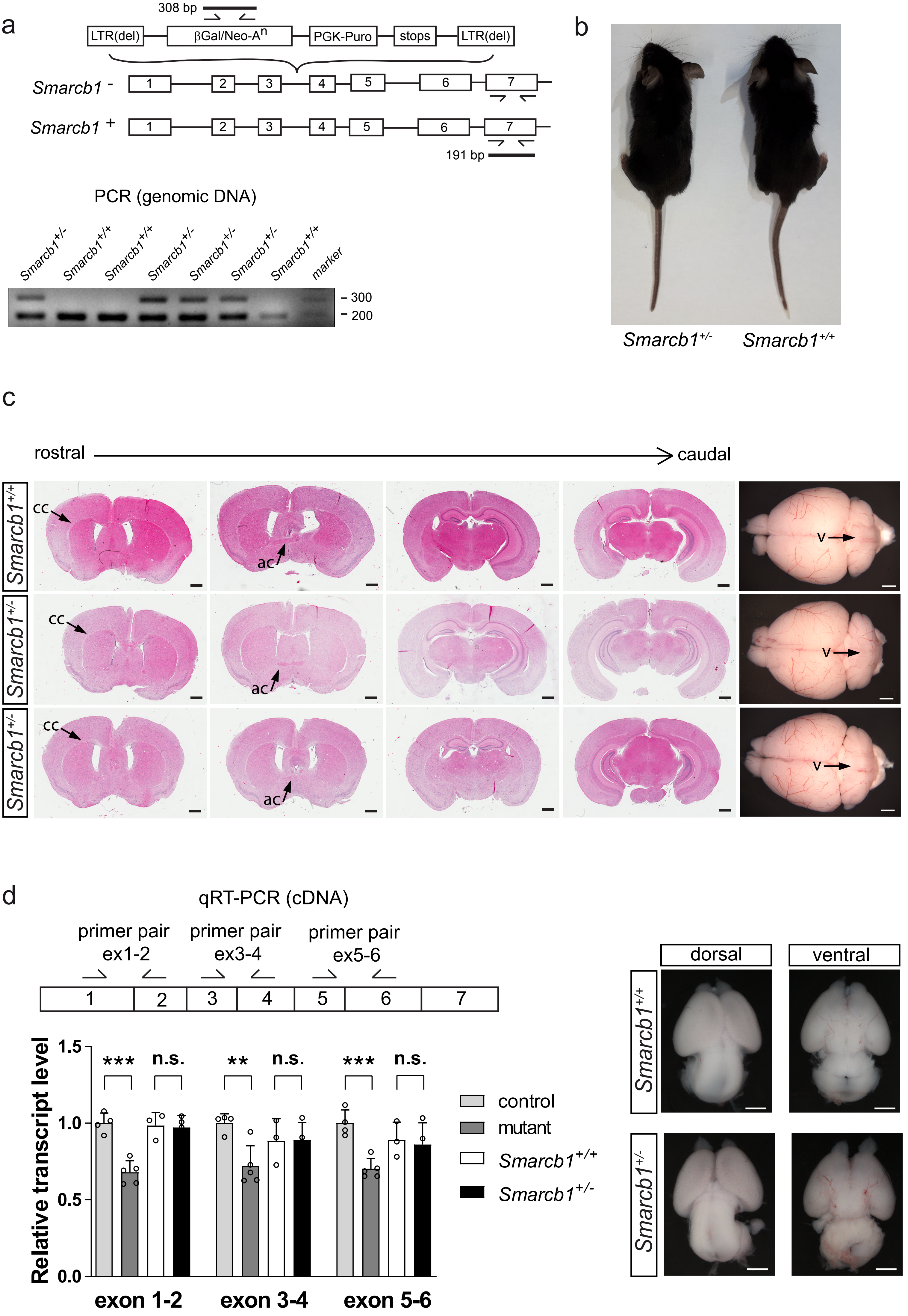
a
308 bp
LTR(del)
βGal/Neo-An
PGK-Puro
stops
LTR(del)
Smarcb1 -
Smarcb1 +
191 bp
PCR (genomic DNA)
Smarcb1+/-
Smarcb1+/+
Smarcb1+/+
Smarcb1+/-
Smarcb1+/-
Smarcb1+/-
Smarcb1+/+
marker
300
200
b
Smarcb1+/-
Smarcb1+/+
c
rostral
caudal
Smarcb1+/+
Smarcb1+/-
Smarcb1+/-
cc
ac
v
d
qRT-PCR (cDNA)
primer pair ex1-2
primer pair ex3-4
primer pair ex5-6
Relative transcript level
***
n.s.
**
n.s.
***
n.s.
control
mutant
Smarcb1+/+
Smarcb1+/-
exon 1-2
exon 3-4
exon 5-6
dorsal
ventral
Smarcb1+/+
Smarcb1+/-

Supplementary Fig. 5

Analyses of *Smarcb1*$^{+/-}$ and *Smarcb1*$^{+/+}$ brains. (**a**) PCR analysis of genomic DNA isolated from E14.5 forebrain tissue of heterozygous conventional *Smarcb1* knockout mice (*Smarcb1*$^{+/-}$) using two primer pairs in one PCR reaction (one amplifying a 308 bp ßGal/Neo-cassette fragment present in the knockout allele, and one amplifying a 191 bp fragment of exon 7). Agarose gel electrophoresis of the PCR products shows the presence of a knockout allele in *Smarcb1*$^{+/-}$ samples. (**b**) Appearance of a *Smarcb1*$^{+/-}$ and a *Smarcb1*$^{+/+}$ littermate animal (age: 3 weeks). (**c**) Representative hematoxylin-eosin-stained coronal sections and brain photographs of one *Smarcb1*$^{+/+}$ and two *Smarcb1*$^{+/-}$ animals (age: 3 weeks). No midline brain abnormalities are present. ac: anterior commissure, cc: corpus callosum, v: vermis. Scale bars: 1 mm. (**d**) *Smarcb1* transcript levels in E14.5 *Smarcb1*$^{+/inv}$ *NesCre*$^{-/-}$ (control)**,** *Smarcb1*$^{+/inv}$ *NesCre*$^{+/-}$ (mutant), *Smarcb1*$^{+/+}$, and *Smarcb1*$^{+/-}$ forebrain tissue determined by quantitative RT-PCR using three different primer pairs, one spanning an exon 1-2 region, one an exon 3-4 region, and the other one an exon 5-6 region. Note that the exon 3-4 spanning primer pair should only amplify cDNA derived from wildtype allele transcripts in *Smarcb1*$^{+/-}$ animals. Tissue from four *Smarcb1*$^{+/inv}$ *NesCre*$^{-/-}$ and five *Smarcb1*$^{+/inv}$ *NesCre*$^{+/-}$ littermates, and from three *Smarcb1*$^{+/+}$ and four *Smarcb1*$^{+/-}$ littermates was used. Bars display the mean with standard deviations. No significantly (n.s.) different *Smarcb1* transcript levels were found between *Smarcb1*$^{+/+}$ and *Smarcb1*$^{+/-}$ samples, whereas levels were significanty reduced in *Smarcb1*$^{+/inv}$ *NesCre*$^{+/-}$ compared with *Smarcb1*$^{+/inv}$ *NesCre*$^{-/-}$ samples (unpaired two-tailed Student's t-test). ** $p \leq 0.01$, *** $p \leq 0.001$. Photographs of E14.5 *Smarcb1*$^{+/+}$ and *Smarcb1*$^{+/-}$ brains are shown. Scale bars: 1 mm. Source data are provided as a Source Data file.

Supplementary Note 1

Individual 1, male, was clinically diagnosed with CSS showing typical features such as nail hypoplasia of the fifth finger and toe, hypoplasia of the distal phalanges of both hands, ID, hypotonia and coarse facial features. A heterozygous *SMARCB1* mutation (*SMARCB1* c.1121G>A p.(Arg374Gln)) was later identified. This patient had short stature, dystrophy and microcephaly[9]. MRI performed at 2 $^{5}/_{12}$ years revealed a short and stubby corpus callosum, and the anterior and hippocampal commissures were not visible (see Fig. 2g). The optic chiasm and optic nerves were small but still within the normal range.

Individual 2, female, showed a global developmental delay, muscular hypotonia, an atrial septal defect, facial dysmorphism and a hypoplastic fifth fingernail on the left hand. The clinical diagnosis of CSS was confirmed by a heterozygous pathogenic variant in *ARID1B* (*ARID1B* c.5961_5964delGAGA p.(Arg1988Ser*fs**32)). Cranial MRI at the age of 3 $^{5}/_{12}$ years in this patient was completely normal.

Individual 3, male, had developmental delay, absent speech and coarse facial features. The suspected diagnosis of CSS was confirmed by a heterozygous pathogenic variant in *ARID1B* (c.3604_3610dupTCCATGG p.(Ala1204Val*fs**8)). Cranial MRI at 2 $^{10}/_{12}$ years revealed a short and stubby corpus callosum and small anterior and hippocampal commissures. The optic chiasm and optic nerves were hypoplastic.

Individual 4, male, was suspected of CSS because of intellectual disability, hypotonia, failure to thrive, and facial dysmorphism. The diagnosis was molecularly confirmed by a heterozygous, pathogenic variant in *ARID1B* (c.5346dupT p.(Lys1783*)). Cranial MRI was performed at ages 2 $^{4}/_{12}$ and 7 $^{2}/_{12}$ years, and revealed multiple abnormalities. Corpus callosum, anterior and hippocampal commissures, and septum pellucidum were all absent (see Fig. 2f,i). Moreover, voluminous choroid plexus tissue was present in both lateral ventricles, and these ventricles showed a colpocephalic configuration (Fig. 2j).

Individual 5, female, showed global intellectual disability, muscular hypotonia, strabismus, sensineuronal deafness, and facial dysmorphisms. CSS was diagnosed by exome sequencing which revealed a heterozygous pathogenic *ARID1B* variant

(c.2191_2192dupAT p.(Pro732Ser_fs_*14)). Cranial MRI at the age of 3 years showed no structural abnormalities.

Individual 6, male, was diagnosed with CSS by exome sequencing, which was performed because of developmental delay, muscular hypotonia and facial dysmorphism and revealed a heterozygous pathogenic variant in *ARID1B* (c.5910_5928del p.(Pro1973Arg_fs_*29)). Cranial MRI was performed at 2 $^{11}/_{12}$ and 3 $^{6}/_{12}$ years, and showed various structural abnormalities. The corpus callosum and septum pellucidum were absent, and the anterior and hippocampal commissures were small. Colpocephaly was noted as well as a reduced size of the hippocampi.

Individual 7, female, showed the typical features of CSS with hypoplastic finger- and toenails and an absent fifth fingernail and facial dysmorphism. She had global intellectual disability, short stature, microcephaly and dystrophy. Genetic testing revealed a heterozygous *de novo* missense variant in *SMARCE1* (c.218A>C p.(Tyr73Ser))[9]. Cranial MRI was performed at 4 months and showed multiple structural abnormalities including a short and thin corpus callosum, a hypoplastic septum pellucidum and small anterior and hippocampal commissures (Fig. 2e,h). The optic chiasm and optic nerves were hypoplastic. Cerebrospinal fluid spaces were moderately enlarged. A Dandy-Walker variant with a large posterior fossa, vermis hypoplasia, and cerebellar dysplasia were also present (asterisk in Fig. 2e).

Individual 8, female, was suspected of CSS because of global developmental delay, growth retardation, microcephaly, and hypoplastic nails. The diagnosis was molecularly confirmed by a heterozygous *de novo* variant in *SMARCB1* (c.1121G>A p.(Arg374Gln)). Cranial MRI was performed at the age of 2 $^{7}/_{12}$ years and showed a short and stubby corpus callosum, a small optic chiasm, unilateral optic nerve hypoplasia and a large intraorbital cyst with optic nerve displacement on the right side. The anterior and hippocampal commissures were not visible.

Individual 9, female, was diagnosed with CSS several weeks after birth because of typical dysmorphic features and severe feeding difficulties requiring tube feeding. She has short stature (-5 standard deviation score, SDS) and had a -3 SDS head cirumference at 5 months of age. No recent head cirumference is available. The diagnosis was molecularly confirmed by a heterozygous pathogenic variant in *SMARCB1* (c.1091_1093delAGA p.(Lys364del)). Cranial MRI was performed at the age of two weeks and showed a short corpus callosum, a Dandy-Walker variant

(large posterior fossa, hypoplastic vermis, hypoplasia of the cerebellum), a hypoplastic optic chiasm, and praechiasmal hypoplastic optic nerve.

Individual 10, female, was small for gestational age, had trachyomalacia for which she needed assisted ventilation, and had severe feeding difficulties. After birth a clinical diagnosis of Cornelia de Lange or Rubinstein-Taybi syndrome was considered. She is -5.5 SDS for height and has a head circumference of -2.5 SDS.

Around the age of 6 years a clinical diagnosis of CSS was made, which was molecularly confirmed by a *de novo* mutation in *SMARCB1* (c.1091_1093delAGA p.(Lys364del)). Cranial MRI was performed at the age of 4 months, and showed small anterior and hippocampal commissures, a short and thin corpus callosum, hypoplastic optic chiasm and optic nerve, an enlarged 4th ventricle, and mild hypoplasia of the cerebellar vermis.

Individual 11, male, had severe feeding difficulties at birth. A clinical diagnosis of Costello syndrome was suggested. He is -4.35 SDS for height; no head cirumference data is available. At 12 years a clinical diagnosis of CSS was made, which was molecularly confirmed by a *de novo* mutation in *SMARCB1* (c.1121G>A p.(Arg374Gln)). Cranial MRI at the age of 3 months showed a short and thin corpus callosum.
