# Supplementary material for: Mutations in SMARCB1 and in other Coffin–Siris syndrome genes lead to various brain midline defects
Source: Nat Commun. 2019 Jul 4;10:2966. doi: 10.1038/s41467-019-10849-y (PMC6609698; doi:10.1038/s41467-019-10849-y)
Supplement: Supplementary file 3 — Reporting Summary [file 41467_2019_10849_MOESM3_ESM.pdf]

nature research

Corresponding author(s): Ulrike A. Nuber

Last updated by author(s): Apr 24, 2019

Reporting Summary

Nature Research wishes to improve the reproducibility of the work that we publish. This form provides structure for consistency and transparency in reporting. For further information on Nature Research policies, see Authors & Referees and the Editorial Policy Checklist.

Statistics

For all statistical analyses, confirm that the following items are present in the figure legend, table legend, main text, or Methods section.

n/a	Confirmed	
☐	☒	The exact sample size (*n*) for each experimental group/condition, given as a discrete number and unit of measurement
☐	☒	A statement on whether measurements were taken from distinct samples or whether the same sample was measured repeatedly
☐	☒	The statistical test(s) used AND whether they are one- or two-sided<br>*Only common tests should be described solely by name; describe more complex techniques in the Methods section.*
☒	☐	A description of all covariates tested
☐	☒	A description of any assumptions or corrections, such as tests of normality and adjustment for multiple comparisons
☐	☒	A full description of the statistical parameters including central tendency (e.g. means) or other basic estimates (e.g. regression coefficient) AND variation (e.g. standard deviation) or associated estimates of uncertainty (e.g. confidence intervals)
☐	☒	For null hypothesis testing, the test statistic (e.g. *F*, *t*, *r*) with confidence intervals, effect sizes, degrees of freedom and *P* value noted<br>*Give P values as exact values whenever suitable.*
☒	☐	For Bayesian analysis, information on the choice of priors and Markov chain Monte Carlo settings
☒	☐	For hierarchical and complex designs, identification of the appropriate level for tests and full reporting of outcomes
☒	☐	Estimates of effect sizes (e.g. Cohen's *d*, Pearson's *r*), indicating how they were calculated

Our web collection on statistics for biologists contains articles on many of the points above.

Software and code

Policy information about availability of computer code

Data collection	Image acquisition of tissue sections stained with hematoxylin and eosin, nuclear fast red, and upon RNA situ hybridization: Aperio CS2 slide scanner (Leica Biosystems)<br>Image acquisition of isolated mouse brains: Stemi SV11 microscope (Zeiss) equipped with a DS-Fi1 camera (Nikon), software: NIS-Elements D3.1 (Nikon)<br>Epifluorescence microscopy: Zeiss Observer D1 or Zeiss Axiovert 200M microscope<br>Quantitative RT-PCR: StepOnePlus Realt-Time PCR System (Applied Biosystems, 2015), software: StepOne Software v2.3, 2012 (Life Technologies Corporation)
Data analysis	Quantitative RT-PCR data analysis: GraphPad Prism 5 (GraphPad software San Diego, CA, USA), Excel 2010 (Microsoft, USA)<br>Image analysis: ImageJ software (https://imagej.nih.gov/ij/index.html; National Institutes of Health, Bethesda, MD, USA)

For manuscripts utilizing custom algorithms or software that are central to the research but not yet described in published literature, software must be made available to editors/reviewers. We strongly encourage code deposition in a community repository (e.g. GitHub). See the Nature Research guidelines for submitting code & software for further information.

Data

Policy information about availability of data

All manuscripts must include a data availability statement. This statement should provide the following information, where applicable:

- Accession codes, unique identifiers, or web links for publicly available datasets
- A list of figures that have associated raw data
- A description of any restrictions on data availability

The source data underlying Figures 1f, 1g, 4b, 4c, 5d, 5e, Supplementary Fig. 1a, Supplementary Fig. 1b, Supplementary Fig. 1c, Supplementary Fig. 1e, Supplementary Fig. 5a, and Supplementary Fig. 5d are provided as Supplementary datasets.

Field-specific reporting

Please select the one below that is the best fit for your research. If you are not sure, read the appropriate sections before making your selection.

☒ Life sciences ☐ Behavioural & social sciences ☐ Ecological, evolutionary & environmental sciences

For a reference copy of the document with all sections, see nature.com/documents/nr-reporting-summary-flat.pdf

Life sciences study design

All studies must disclose on these points even when the disclosure is negative.

Sample size	We did not perform a sample size calculation a priori and collected as many samples as possible. For analyses of embryonic mice, we used mutant and control animals from the same litter, which limits the number of animals per group.
Data exclusions	We did not exclude any data.
Replication	Experimental results were reproduced either by repeat experiments using identical samples and/or by applying a minimum of three biological replicates.
Randomization	There was no need for a systematic randomization of mouse samples. The sample order within a set of experiments was independent of the genotype.
Blinding	Mouse samples were assigned continuous numbers without explicit genotype information. No explicit blinding was performed.

Reporting for specific materials, systems and methods

We require information from authors about some types of materials, experimental systems and methods used in many studies. Here, indicate whether each material, system or method listed is relevant to your study. If you are not sure if a list item applies to your research, read the appropriate section before selecting a response.

Materials & experimental systems

n/a	Involved in the study	
☐	☒	Antibodies
☐	☐	Eukaryotic cell lines
☐	☐	Palaeontology
☐	☒	Animals and other organisms
☐	☐	Human research participants
☐	☒	Clinical data

Methods

n/a	Involved in the study	
☐	☐	ChIP-seq
☐	☐	Flow cytometry
☐	☒	MRI-based neuroimaging

Antibodies

Antibodies used	Ctip2 (Abcam, ab18465, lot 25B6), Gfap (Dako, Z0334, lot 00073720), GLAST (Abcam, ab416-200, lot 191142), Ki67 (Abcam, ab16667, lot 41454A), Laminin (Sigma, L9393), Nrp1 (R&D, AF566, lot ETH0918031), Otx2 (R&D, AF1979, lot KNO0615111), Satb2 (Abcam, ab69995), SMARCB1 (CST, 91735, lot 91735S), Sox2 (Santa Cruz Biotechnology, sc-17320, lotG2516), Tbr2 (Abcam, ab23345, lot GR306193-1). Secondary antibodies coupled to Cy3, AF488, and AF647 were from Dianova.
Validation	Catalogue numbers are provided for validation information on the manufacturers´ websites.<br>The specificity of the following antibodies was validated by us based on immunostaining patterns on mouse tissue sections: Ctip2, Gfap, GLAST, Laminin, Nrp1, Otx2, Satb2, Sox2, Tbr2. We validated the specificity of the Smarcb1 antibody by immunoblotting.

Eukaryotic cell lines

Policy information about cell lines

Cell line source(s)	*State the source of each cell line used.*
Authentication	*Describe the authentication procedures for each cell line used OR declare that none of the cell lines used were authenticated.*
Mycoplasma contamination	*Confirm that all cell lines tested negative for mycoplasma contamination OR describe the results of the testing for mycoplasma contamination OR declare that the cell lines were not tested for mycoplasma contamination.*

Commonly misidentified lines
(See ICLAC register)

Name any commonly misidentified cell lines used in the study and provide a rationale for their use.

Palaeontology

Specimen provenance

Provide provenance information for specimens and describe permits that were obtained for the work (including the name of the issuing authority, the date of issue, and any identifying information).

Specimen deposition

Indicate where the specimens have been deposited to permit free access by other researchers.

Dating methods

If new dates are provided, describe how they were obtained (e.g. collection, storage, sample pretreatment and measurement), where they were obtained (i.e. lab name), the calibration program and the protocol for quality assurance OR state that no new dates are provided.

☐ Tick this box to confirm that the raw and calibrated dates are available in the paper or in Supplementary Information.

Animals and other organisms

Policy information about studies involving animals; ARRIVE guidelines recommended for reporting animal research

Laboratory animals

Mouse strains used (embryonic and postnatal stages):
Smarcb1+/inv mice (originally termed Snf5+/inv)
Smarcb1+/- mice (originally termed Ini1-heterozygous mice)
NesCre+/- mice
C57BL/6N

Wild animals

Provide details on animals observed in or captured in the field; report species, sex and age where possible. Describe how animals were caught and transported and what happened to captive animals after the study (if killed, explain why and describe method; if released, say where and when) OR state that the study did not involve wild animals.

Field-collected samples

For laboratory work with field-collected samples, describe all relevant parameters such as housing, maintenance, temperature, photoperiod and end-of-experiment protocol OR state that the study did not involve samples collected from the field.

Ethics oversight

All animal procedures were approved by the local authorities (Regierungspräsidium Darmstadt, Hesse, Germany)

Note that full information on the approval of the study protocol must also be provided in the manuscript.

Human research participants

Policy information about studies involving human research participants

Population characteristics

Describe the covariate-relevant population characteristics of the human research participants (e.g. age, gender, genotypic information, past and current diagnosis and treatment categories). If you filled out the behavioural & social sciences study design questions and have nothing to add here, write "See above."

Recruitment

Describe how participants were recruited. Outline any potential self-selection bias or other biases that may be present and how these are likely to impact results.

Ethics oversight

Identify the organization(s) that approved the study protocol.

Note that full information on the approval of the study protocol must also be provided in the manuscript.

Clinical data

Policy information about clinical studies

All manuscripts should comply with the ICMJE guidelines for publication of clinical research and a completed CONSORT checklist must be included with all submissions.

Clinical trial registration

Provide the trial registration number from ClinicalTrials.gov or an equivalent agency.

Study protocol

Note where the full trial protocol can be accessed OR if not available, explain why.

Data collection

Informed consent was obtained for publication of MRI scans and clinical data.

Outcomes

Describe how you pre-defined primary and secondary outcome measures and how you assessed these measures.

ChIP-seq

Data deposition

☐ Confirm that both raw and final processed data have been deposited in a public database such as GEO.

☐ Confirm that you have deposited or provided access to graph files (e.g. BED files) for the called peaks.

Data access links<br>*May remain private before publication.*	*For "Initial submission" or "Revised version" documents, provide reviewer access links. For your "Final submission" document, provide a link to the deposited data.*
Files in database submission	*Provide a list of all files available in the database submission.*
Genome browser session<br>(e.g. UCSC)	*Provide a link to an anonymized genome browser session for "Initial submission" and "Revised version" documents only, to enable peer review. Write "no longer applicable" for "Final submission" documents.*

Methodology

Replicates	*Describe the experimental replicates, specifying number, type and replicate agreement.*
Sequencing depth	*Describe the sequencing depth for each experiment, providing the total number of reads, uniquely mapped reads, length of reads and whether they were paired- or single-end.*
Antibodies	*Describe the antibodies used for the ChIP-seq experiments; as applicable, provide supplier name, catalog number, clone name, and lot number.*
Peak calling parameters	*Specify the command line program and parameters used for read mapping and peak calling, including the ChIP, control and index files used.*
Data quality	*Describe the methods used to ensure data quality in full detail, including how many peaks are at FDR 5% and above 5-fold enrichment.*
Software	*Describe the software used to collect and analyze the ChIP-seq data. For custom code that has been deposited into a community repository, provide accession details.*

Flow Cytometry

Plots

Confirm that:

☐ The axis labels state the marker and fluorochrome used (e.g. CD4-FITC).

☐ The axis scales are clearly visible. Include numbers along axes only for bottom left plot of group (a 'group' is an analysis of identical markers).

☐ All plots are contour plots with outliers or pseudocolor plots.

☐ A numerical value for number of cells or percentage (with statistics) is provided.

Methodology

Sample preparation	*Describe the sample preparation, detailing the biological source of the cells and any tissue processing steps used.*
Instrument	*Identify the instrument used for data collection, specifying make and model number.*
Software	*Describe the software used to collect and analyze the flow cytometry data. For custom code that has been deposited into a community repository, provide accession details.*
Cell population abundance	*Describe the abundance of the relevant cell populations within post-sort fractions, providing details on the purity of the samples and how it was determined.*
Gating strategy	*Describe the gating strategy used for all relevant experiments, specifying the preliminary FSC/SSC gates of the starting cell population, indicating where boundaries between "positive" and "negative" staining cell populations are defined.*

☐ Tick this box to confirm that a figure exemplifying the gating strategy is provided in the Supplementary Information.

Magnetic resonance imaging

Experimental design

Design type	Not applicable.

Design specifications | Not applicable.

Behavioral performance measures | Not performed.

Acquisition

Imaging type(s) | Structural, functional

Field strength | 1,5 T; T1/T2

Sequence & imaging parameters | T2w-TSE transversal, T2w-TIRM transversal, DWI-EPI transversal, arterial/venous ToF, MRA transversal and coronal, SWI transversal; TSE, FLAIR, DWI, 3d ns IR, TIR, cis, in T1 and T2

Area of acquisition | *State whether a whole brain scan was used OR define the area of acquisition, describing how the region was determined.*

Diffusion MRI ☐ Used ☐ Not used

Preprocessing

Preprocessing software | *Provide detail on software version and revision number and on specific parameters (model/functions, brain extraction, segmentation, smoothing kernel size, etc.).*

Normalization | *If data were normalized/standardized, describe the approach(es): specify linear or non-linear and define image types used for transformation OR indicate that data were not normalized and explain rationale for lack of normalization.*

Normalization template | *Describe the template used for normalization/transformation, specifying subject space or group standardized space (e.g. original Talairach, MNI305, ICBM152) OR indicate that the data were not normalized.*

Noise and artifact removal | *Describe your procedure(s) for artifact and structured noise removal, specifying motion parameters, tissue signals and physiological signals (heart rate, respiration).*

Volume censoring | *Define your software and/or method and criteria for volume censoring, and state the extent of such censoring.*

Statistical modeling & inference

Model type and settings | Not applicable.

Effect(s) tested | None.

Specify type of analysis: ☐ Whole brain ☐ ROI-based ☐ Both

Statistic type for inference (See Eklund et al. 2016) | *Specify voxel-wise or cluster-wise and report all relevant parameters for cluster-wise methods.*

Correction | *Describe the type of correction and how it is obtained for multiple comparisons (e.g. FWE, FDR, permutation or Monte Carlo).*

Models & analysis

n/a	Involved in the study
☒	☐ Functional and/or effective connectivity
☒	☐ Graph analysis
☒	☐ Multivariate modeling or predictive analysis
